# (2,7-Dimeth­oxy­naphthalen-1-yl)(4-meth­oxy­phen­yl)methanone

**DOI:** 10.1107/S1600536813003218

**Published:** 2013-02-09

**Authors:** Kosuke Sasagawa, Rei Sakamoto, Daichi Hijikata, Akiko Okamoto, Noriyuki Yonezawa

**Affiliations:** a Department of Organic and Polymer Materials Chemistry, Tokyo University of Agriculture & Technology, Koganei, Tokyo 184-8588, Japan

## Abstract

In the mol­ecule of the title compound, C_20_H_18_O_4_, the dihedral angle between the naphthalene ring system and the benzene ring is 81.74 (5)°. An inter­molecular C—H⋯O inter­action is formed between an H atom at the 6-position of the naphthalene ring and the O atom of the meth­oxy group at the 7-position.

## Related literature

For formation reactions of aroylated naphthalene compounds *via* electrophilic aromatic substitution of naphthalene deriv­atives, see: Okamoto & Yonezawa (2009 ▶); Okamoto *et al.* (2011 ▶). For the structures of closely related compounds, see: Nakaema *et al.* (2008 ▶); Hijikata *et al.* (2010 ▶); Kato *et al.* (2010 ▶); Tsumuki *et al.* (2011 ▶, 2012 ▶).
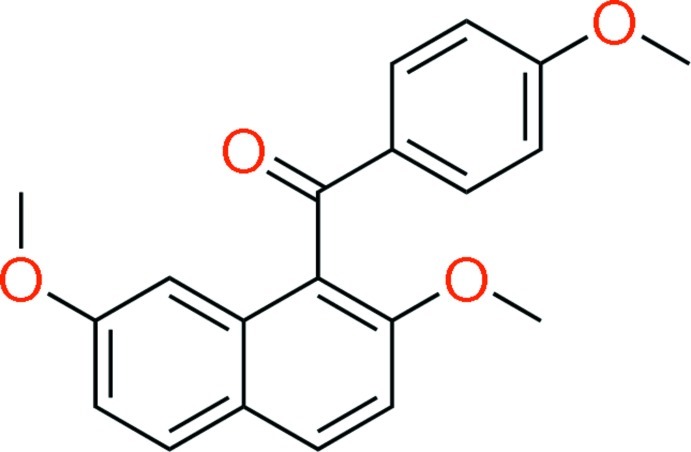


## Experimental

### 

#### Crystal data

C_20_H_18_O_4_
*M* *_r_* = 322.34
Monoclinic, 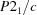

*a* = 14.9638 (2) Å
*b* = 7.9191 (1) Å
*c* = 13.6394 (2) Å
β = 92.56°
*V* = 1614.64 (4) Å^3^
*Z* = 4
Cu *K*α radiation
μ = 0.75 mm^−1^
*T* = 193 K
0.60 × 0.40 × 0.30 mm

#### Data collection

Rigaku R-AXIS RAPID diffractometer
Absorption correction: numerical (*NUMABS*; Higashi, 1999 ▶) *T*_min_ = 0.662, *T*_max_ = 0.806
27665 measured reflections
2957 independent reflections
2701 reflections with *I* > 2σ(*I*)
*R*_int_ = 0.051

#### Refinement

*R*[*F* ^2^ > 2σ(*F* ^2^)] = 0.038
*wR*(*F* ^2^) = 0.106
*S* = 1.05
2957 reflections
221 parameters
H-atom parameters constrained
Δρ_max_ = 0.24 e Å^−3^
Δρ_min_ = −0.18 e Å^−3^

### 

Data collection: *PROCESS-AUTO* (Rigaku, 1998 ▶); cell refinement: *PROCESS-AUTO*; data reduction: *CrystalStructure* (Rigaku, 2010 ▶); program(s) used to solve structure: *Il Milione* (Burla *et al.*, 2007 ▶); program(s) used to refine structure: *SHELXL97* (Sheldrick, 2008 ▶); molecular graphics: *ORTEPIII* (Burnett & Johnson, 1996 ▶); software used to prepare material for publication: *SHELXL97*.

## Supplementary Material

Crystal structure: contains datablock(s) I, global. DOI: 10.1107/S1600536813003218/pk2464sup1.cif

Structure factors: contains datablock(s) I. DOI: 10.1107/S1600536813003218/pk2464Isup2.hkl

Supplementary material file. DOI: 10.1107/S1600536813003218/pk2464Isup3.cml

Additional supplementary materials:  crystallographic information; 3D view; checkCIF report

## Figures and Tables

**Table 1:** Hydrogen-bond geometry (Å, °)

*D*—H⋯*A*	*D*—H	H⋯*A*	*D*⋯*A*	*D*—H⋯*A*
C6—H6⋯O3^i^	0.95	2.51	3.4592 (16)	178

Symmetry code: (i) 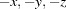.

**Fig. 1.**
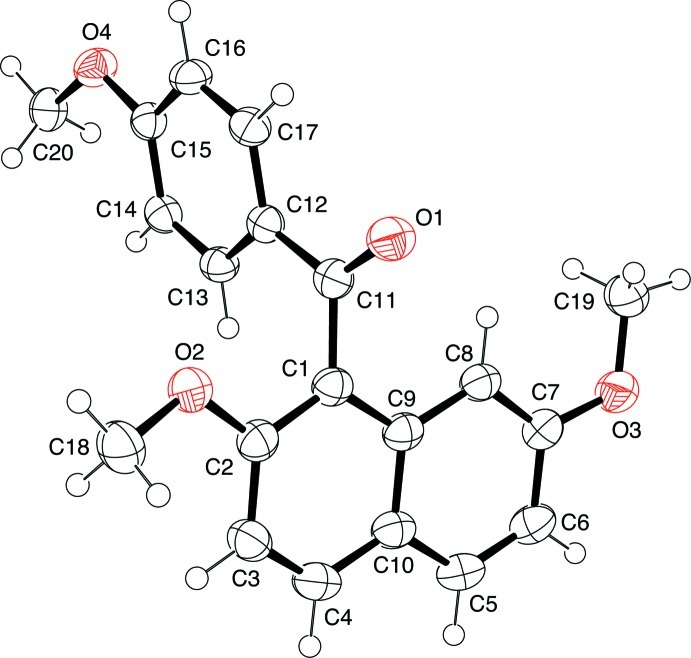
Molecular structure with the atom-labeling scheme and displacement ellipsoids drawn at the 50% probability level.

**Fig. 2.**
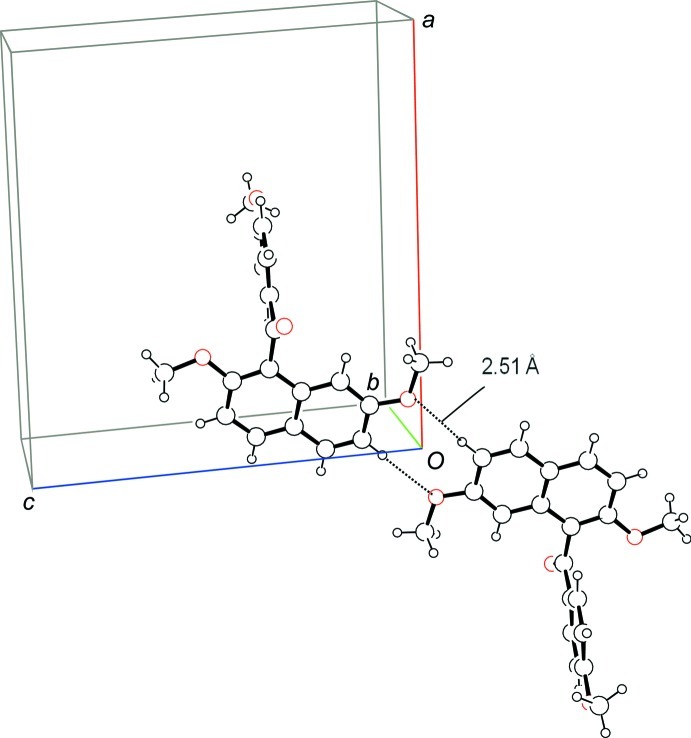
Intermolecular C—H···O interactions between H5 and O3 [symmetry code: -x, -y, -z] along the c axis (dashed lines).

**Fig. 3.**
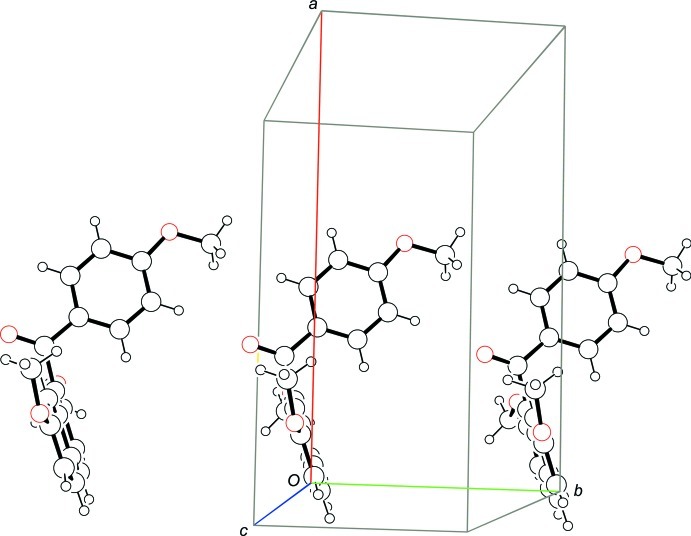
Alignment of the naphthalene rings along b axis.

## supplementary crystallographic information

### Comment

In the course of our study on selective electrophilic aromatic aroylation of the naphthalene ring core, 1-aroylnaphthalene and 1,8-diaroylnaphthalene compounds have proved to be formed regioselectively by the aid of a suitable acidic mediator (Okamoto & Yonezawa, 2009, Okamoto *et al.*, 2011). Recently, we have reported the X-ray crystal structures of 1,8-diaroylated 2,7-dimethoxynaphthalene derivatives such as 1,8-dibenzoyl-2,7-dimethoxynaphthalene (Nakaema *et al.*, 2008) and [2,7-dimethoxy-8-(2-naphthoyl)-naphthalen-1-yl](naphthalen-2-yl)-methanone [1,8-bis(2-naphthoyl)-2,7-dimethoxynaphthalene] (Tsumuki *et al.*, 2011). The aroyl groups in the 1,8-diaroylnaphthalene compounds are almost perpendicular to the naphthalene rings and oriented in opposite directions (*anti*-orientation). On the other hand, we have also clarified the minor structure of the 1,8-diaroylnaphthalene derivatives, which the two aroyl groups are oriented in same direction (*syn*-orientation), [2,7-dimethoxy-1,8-bis(4-phenoxybenzoyl)naphthalene; Hijikata *et al.*, 2010]. Moreover, we have reported crystal structures of 1-aroylnapthalene compounds such as (2,7-dimethoxynaphthalen-1-yl)-(phenyl)methanone (1-benzoyl-2,7-dimethoxynaphthalene) (Kato *et al.*, 2010) and 2,7-dimethoxy-1-(2-naphthoyl)naphthalene (Tsumuki *et al.*, 2012). They have essentially same non-coplanar structure as the homologous 1,8-diaroylnaphthalenes.

As a part of our ongoing studies on the molecular structures of these kinds of homologous molecules, the X-ray crystal structure of the title compound, 1-aroylated naphthalene bearing methoxy groups on aroyl group, is discussed in this article.

The molecular structure of the title compound is displayed in Fig. 1. The dihedral angle between the best planes of the phenyl ring and the naphthalene ring is 81.74 (5) °.

The dihedral angle between the naphthalene ring system and the bridging ketonic carbonyl C—C(=O)—C plane is larger than that between the phenyl ring and the bridging carbonyl plane [74.77 (6)° *versus*. 13.27 (6) °].

In the molecular packing, C—H···O interactions between the aromatic hydrogen atoms at the 6-position of the naphthalene ring and the methoxy oxygen atoms at the 7-position are observed (C5—H5···O3 = 2.51 Å; symmetry code: -*x*, -*y*, -*z*; Fig. 2). Moreover, the naphthalene rings are aligned in parallel to each other along *b* axis (Fig. 3).

### Experimental

4-Methoxybenzoyl chloride (11.0 mmol, 1.96 g), aluminium chloride (11.0 mmol, 1.47 g) and methylene chloride (25.0 ml) were placed into a 100 ml flask, followed by stirring at 273 K. To the reaction mixture thus obtained, 2,7-dimethoxynaphthalene (10.0 mmol, 1.88 g) was added. The reaction mixture was poured into ice-cold water (100 ml) after it had been stirred for 6 h at 273 K. The aqueous layer was extracted with CHCl_3_ (20 ml × 3). The combined extracts were washed with 2*M* aqueous NaOH followed by washing with brine. The extracts thus obtained were dried over anhydrous MgSO_4_. The solvent was removed under reduced pressure to give a cake. The crude product was purified by recrystallization from methanol (yield 44%). Colorless platelet single crystals suitable for X-ray diffraction were obtained by repeated crystallization from methanol.

Spectroscopic Data:

^1^H NMR δ (300 MHz, CDCl_3_): 3.70 (3*H*, s), 3.79 (3*H*, s), 3.83 (3*H*, s), 6.78 (1*H*, d, *J* = 2.4 Hz), 6.89 (2*H*, d, *J* = 9.0 Hz), 7.00 (1*H*, dd, *J* = 9.0, 2.4 Hz), 7.15 (1*H*, d, *J* = 9.0 Hz), 7.70 (1*H*, d, *J* = 9.0 Hz), 7.83 (3*H*, d, *J* = 9.0 Hz) p.p.m. ^13^C NMR δ (75 MHz, CDCl_3_): 55.14, 55.41, 56.36, 102.17, 110.28, 113.73, 116.98, 122.15, 124.34, 129.56, 130.61, 131.09, 131.98, 132.98, 154.60, 158.69, 163.81, 196.54 p.p.m. IR (KBr): 1659 (C=O), 1624, 1599, 1510 (Ar), 1251 (OMe) cm^-1^ HRMS (m/*z*): [*M*+H]^+^ calcd. for C_20_H_19_O_4_, 323.1283, found, 323.1332 m.p. = 368.5–368.9 K

### Refinement

All H atoms were found in a difference map and were subsequently refined as riding atoms, with C—H = 0.95 (aromatic) and 0.98 (methyl) Å with *U*_ĩso_(H) = 1.2 *U*_eq_(C).

### Crystal data

**Table:**

C_20_H_18_O_4_	*F*(000) = 680
*M**_r_* = 322.34	*D*_x_ = 1.326 Mg m^−^^3^
Monoclinic, *P*2_1_/*c*	Cu *K*α radiation, λ = 1.5418 Å
Hall symbol: -P 2ybc	Cell parameters from 25377 reflections
*a* = 14.9638 (2) Å	θ = 3.0–68.2°
*b* = 7.9191 (1) Å	µ = 0.75 mm^−^^1^
*c* = 13.6394 (2) Å	*T* = 193 K
β = 92.56°	Block, colorless
*V* = 1614.64 (4) Å^3^	0.60 × 0.40 × 0.30 mm
*Z* = 4	

### Data collection

**Table:**

Rigaku R-AXIS RAPID diffractometer	2957 independent reflections
Radiation source: fine-focus sealed tube	2701 reflections with *I* > 2σ(*I*)
Graphite monochromator	*R*_int_ = 0.051
Detector resolution: 10.000 pixels mm^-1^	θ_max_ = 68.2°, θ_min_ = 3.0°
ω scans	*h* = −17→18
Absorption correction: numerical (*NUMABS*; Higashi, 1999)	*k* = −9→9
*T*_min_ = 0.662, *T*_max_ = 0.806	*l* = −16→16
27665 measured reflections	

### Refinement

**Table:**

Refinement on *F*^2^	Secondary atom site location: difference Fourier map
Least-squares matrix: full	Hydrogen site location: inferred from neighbouring sites
*R*[*F*^2^ > 2σ(*F*^2^)] = 0.038	H-atom parameters constrained
*wR*(*F*^2^) = 0.106	*w* = 1/[σ^2^(*F*_o_^2^) + (0.0622*P*)^2^ + 0.323*P*] where *P* = (*F*_o_^2^ + 2*F*_c_^2^)/3
*S* = 1.05	(Δ/σ)_max_ < 0.001
2957 reflections	Δρ_max_ = 0.24 e Å^−^^3^
221 parameters	Δρ_min_ = −0.18 e Å^−^^3^
0 restraints	Extinction correction: *SHELXL97* (Sheldrick, 2008), Fc^*^=kFc[1+0.001xFc^2^λ^3^/sin(2θ)]^-1/4^
Primary atom site location: structure-invariant direct methods	Extinction coefficient: 0.0093 (6)

### Special details

**Table:**

Geometry. All e.s.d.'s (except the e.s.d. in the dihedral angle between two l.s. planes) are estimated using the full covariance matrix. The cell e.s.d.'s are taken into account individually in the estimation of e.s.d.'s in distances, angles and torsion angles; correlations between e.s.d.'s in cell parameters are only used when they are defined by crystal symmetry. An approximate (isotropic) treatment of cell e.s.d.'s is used for estimating e.s.d.'s involving l.s. planes.
Refinement. Refinement of *F*^2^ against ALL reflections. The weighted *R*-factor *wR* and goodness of fit *S* are based on *F*^2^, conventional *R*-factors *R* are based on *F*, with *F* set to zero for negative *F*^2^. The threshold expression of *F*^2^ > σ(*F*^2^) is used only for calculating *R*-factors(gt) *etc*. and is not relevant to the choice of reflections for refinement. *R*-factors based on *F*^2^ are statistically about twice as large as those based on *F*, and *R*- factors based on ALL data will be even larger.

### Fractional atomic coordinates and isotropic or equivalent isotropic displacement parameters (Å^2^)

**Table:**

	*x*	*y*	*z*	*U*_iso_*/*U*_eq_	
O1	0.34025 (6)	−0.17441 (11)	0.36039 (7)	0.0431 (3)	
O2	0.26300 (6)	0.04715 (12)	0.55470 (6)	0.0428 (3)	
O3	0.12880 (6)	−0.05902 (13)	0.04035 (7)	0.0463 (3)	
O4	0.58557 (5)	0.47580 (11)	0.37408 (7)	0.0392 (2)	
C1	0.22392 (8)	0.02329 (15)	0.38741 (9)	0.0324 (3)	
C2	0.19845 (8)	0.06572 (15)	0.48029 (9)	0.0355 (3)	
C3	0.11163 (8)	0.12450 (17)	0.49646 (10)	0.0403 (3)	
H3	0.0950	0.1532	0.5608	0.048*	
C4	0.05162 (8)	0.13973 (17)	0.41872 (10)	0.0412 (3)	
H4	−0.0063	0.1833	0.4294	0.049*	
C5	0.01000 (8)	0.10274 (18)	0.24246 (10)	0.0423 (3)	
H5	−0.0480	0.1466	0.2526	0.051*	
C6	0.03049 (8)	0.05121 (18)	0.15138 (10)	0.0430 (3)	
H6	−0.0132	0.0565	0.0988	0.052*	
C7	0.11745 (8)	−0.01061 (17)	0.13501 (9)	0.0373 (3)	
C8	0.18197 (8)	−0.01881 (15)	0.20971 (9)	0.0342 (3)	
H8	0.2404	−0.0578	0.1970	0.041*	
C9	0.16091 (8)	0.03151 (15)	0.30639 (9)	0.0325 (3)	
C10	0.07321 (8)	0.09269 (16)	0.32321 (9)	0.0364 (3)	
C11	0.31989 (8)	−0.02724 (15)	0.37353 (8)	0.0318 (3)	
C12	0.38717 (7)	0.11016 (15)	0.37531 (8)	0.0294 (3)	
C13	0.36306 (7)	0.28000 (15)	0.36920 (8)	0.0311 (3)	
H13	0.3015	0.3094	0.3664	0.037*	
C14	0.42667 (8)	0.40714 (15)	0.36708 (8)	0.0317 (3)	
H14	0.4090	0.5221	0.3614	0.038*	
C15	0.51697 (7)	0.36349 (15)	0.37345 (8)	0.0311 (3)	
C16	0.54233 (7)	0.19433 (16)	0.38133 (9)	0.0348 (3)	
H16	0.6039	0.1652	0.3868	0.042*	
C17	0.47838 (8)	0.06988 (15)	0.38119 (9)	0.0333 (3)	
H17	0.4963	−0.0451	0.3851	0.040*	
C18	0.23465 (11)	0.0506 (2)	0.65301 (10)	0.0510 (4)	
H18A	0.1872	−0.0331	0.6605	0.061*	
H18B	0.2118	0.1633	0.6679	0.061*	
H18C	0.2854	0.0239	0.6982	0.061*	
C19	0.21651 (9)	−0.1007 (2)	0.01257 (10)	0.0474 (3)	
H19A	0.2387	−0.1976	0.0511	0.057*	
H19B	0.2562	−0.0038	0.0248	0.057*	
H19C	0.2151	−0.1293	−0.0574	0.057*	
C20	0.56375 (9)	0.65140 (16)	0.37488 (9)	0.0381 (3)	
H20A	0.6189	0.7181	0.3815	0.046*	
H20B	0.5263	0.6754	0.4303	0.046*	
H20C	0.5311	0.6812	0.3134	0.046*	

### Atomic displacement parameters (Å^2^)

**Table:**

	*U*^11^	*U*^22^	*U*^33^	*U*^12^	*U*^13^	*U*^23^
O1	0.0377 (5)	0.0318 (5)	0.0599 (6)	0.0008 (4)	0.0037 (4)	−0.0008 (4)
O2	0.0388 (5)	0.0573 (6)	0.0324 (5)	−0.0005 (4)	0.0015 (4)	−0.0039 (4)
O3	0.0376 (5)	0.0627 (6)	0.0380 (5)	0.0012 (4)	−0.0039 (4)	−0.0055 (4)
O4	0.0325 (4)	0.0321 (5)	0.0529 (5)	−0.0025 (3)	0.0004 (4)	0.0000 (4)
C1	0.0305 (6)	0.0302 (6)	0.0367 (6)	−0.0026 (5)	0.0038 (5)	0.0014 (5)
C2	0.0346 (6)	0.0349 (7)	0.0372 (6)	−0.0039 (5)	0.0023 (5)	−0.0005 (5)
C3	0.0399 (7)	0.0416 (7)	0.0403 (7)	−0.0006 (5)	0.0102 (5)	−0.0040 (5)
C4	0.0333 (6)	0.0401 (7)	0.0510 (7)	0.0027 (5)	0.0099 (5)	0.0013 (6)
C5	0.0283 (6)	0.0479 (8)	0.0506 (7)	0.0013 (5)	0.0022 (5)	0.0041 (6)
C6	0.0323 (6)	0.0517 (8)	0.0446 (7)	−0.0013 (5)	−0.0045 (5)	0.0040 (6)
C7	0.0348 (6)	0.0389 (7)	0.0380 (6)	−0.0036 (5)	−0.0003 (5)	0.0011 (5)
C8	0.0292 (6)	0.0339 (6)	0.0396 (7)	−0.0014 (5)	0.0021 (5)	0.0011 (5)
C9	0.0302 (6)	0.0288 (6)	0.0385 (6)	−0.0028 (4)	0.0028 (5)	0.0028 (5)
C10	0.0303 (6)	0.0351 (7)	0.0442 (7)	−0.0013 (5)	0.0046 (5)	0.0028 (5)
C11	0.0328 (6)	0.0345 (7)	0.0281 (6)	0.0024 (5)	0.0002 (4)	0.0020 (5)
C12	0.0306 (6)	0.0310 (6)	0.0264 (5)	0.0016 (4)	0.0009 (4)	0.0011 (4)
C13	0.0285 (5)	0.0350 (6)	0.0298 (5)	0.0042 (5)	0.0009 (4)	0.0014 (5)
C14	0.0351 (6)	0.0294 (6)	0.0306 (6)	0.0039 (5)	0.0008 (4)	0.0010 (5)
C15	0.0314 (6)	0.0329 (6)	0.0289 (5)	−0.0007 (5)	0.0008 (4)	−0.0010 (5)
C16	0.0271 (6)	0.0351 (7)	0.0419 (7)	0.0044 (5)	0.0000 (5)	0.0002 (5)
C17	0.0339 (6)	0.0294 (6)	0.0365 (6)	0.0050 (5)	0.0010 (5)	0.0015 (5)
C18	0.0548 (8)	0.0635 (10)	0.0348 (7)	0.0035 (7)	0.0044 (6)	−0.0049 (6)
C19	0.0430 (7)	0.0600 (9)	0.0393 (7)	0.0039 (6)	0.0021 (6)	−0.0061 (6)
C20	0.0441 (7)	0.0321 (7)	0.0379 (6)	−0.0042 (5)	0.0004 (5)	0.0006 (5)

### Geometric parameters (Å, º)

**Table:**

O1—C11	1.2199 (15)	C8—H8	0.9500
O2—C2	1.3773 (15)	C9—C10	1.4270 (17)
O2—C18	1.4245 (16)	C11—C12	1.4819 (16)
O3—C7	1.3648 (15)	C12—C13	1.3941 (17)
O3—C19	1.4210 (16)	C12—C17	1.4004 (16)
O4—C15	1.3580 (14)	C13—C14	1.3867 (17)
O4—C20	1.4286 (15)	C13—H13	0.9500
C1—C2	1.3807 (17)	C14—C15	1.3936 (16)
C1—C9	1.4216 (17)	C14—H14	0.9500
C1—C11	1.5108 (16)	C15—C16	1.3951 (17)
C2—C3	1.4066 (17)	C16—C17	1.3736 (17)
C3—C4	1.3638 (19)	C16—H16	0.9500
C3—H3	0.9500	C17—H17	0.9500
C4—C10	1.4063 (18)	C18—H18A	0.9800
C4—H4	0.9500	C18—H18B	0.9800
C5—C6	1.3556 (19)	C18—H18C	0.9800
C5—C10	1.4217 (18)	C19—H19A	0.9800
C5—H5	0.9500	C19—H19B	0.9800
C6—C7	1.4172 (18)	C19—H19C	0.9800
C6—H6	0.9500	C20—H20A	0.9800
C7—C8	1.3735 (17)	C20—H20B	0.9800
C8—C9	1.4262 (17)	C20—H20C	0.9800
			
C2—O2—C18	117.58 (10)	C13—C12—C17	118.17 (11)
C7—O3—C19	118.23 (10)	C13—C12—C11	122.25 (10)
C15—O4—C20	117.68 (9)	C17—C12—C11	119.57 (11)
C2—C1—C9	120.09 (11)	C14—C13—C12	121.71 (10)
C2—C1—C11	118.84 (10)	C14—C13—H13	119.1
C9—C1—C11	121.05 (10)	C12—C13—H13	119.1
O2—C2—C1	115.90 (10)	C13—C14—C15	118.89 (11)
O2—C2—C3	122.82 (11)	C13—C14—H14	120.6
C1—C2—C3	121.28 (12)	C15—C14—H14	120.6
C4—C3—C2	119.21 (12)	O4—C15—C14	124.64 (11)
C4—C3—H3	120.4	O4—C15—C16	115.18 (10)
C2—C3—H3	120.4	C14—C15—C16	120.17 (11)
C3—C4—C10	121.75 (11)	C17—C16—C15	120.12 (11)
C3—C4—H4	119.1	C17—C16—H16	119.9
C10—C4—H4	119.1	C15—C16—H16	119.9
C6—C5—C10	121.54 (12)	C16—C17—C12	120.91 (11)
C6—C5—H5	119.2	C16—C17—H17	119.5
C10—C5—H5	119.2	C12—C17—H17	119.5
C5—C6—C7	119.69 (12)	O2—C18—H18A	109.5
C5—C6—H6	120.2	O2—C18—H18B	109.5
C7—C6—H6	120.2	H18A—C18—H18B	109.5
O3—C7—C8	125.13 (11)	O2—C18—H18C	109.5
O3—C7—C6	113.58 (11)	H18A—C18—H18C	109.5
C8—C7—C6	121.29 (12)	H18B—C18—H18C	109.5
C7—C8—C9	119.71 (11)	O3—C19—H19A	109.5
C7—C8—H8	120.1	O3—C19—H19B	109.5
C9—C8—H8	120.1	H19A—C19—H19B	109.5
C1—C9—C8	122.65 (11)	O3—C19—H19C	109.5
C1—C9—C10	118.25 (11)	H19A—C19—H19C	109.5
C8—C9—C10	119.10 (11)	H19B—C19—H19C	109.5
C4—C10—C5	122.03 (11)	O4—C20—H20A	109.5
C4—C10—C9	119.33 (12)	O4—C20—H20B	109.5
C5—C10—C9	118.64 (11)	H20A—C20—H20B	109.5
O1—C11—C12	122.00 (11)	O4—C20—H20C	109.5
O1—C11—C1	121.09 (11)	H20A—C20—H20C	109.5
C12—C11—C1	116.90 (10)	H20B—C20—H20C	109.5
			
C18—O2—C2—C1	−165.55 (12)	C6—C5—C10—C9	1.9 (2)
C18—O2—C2—C3	14.60 (18)	C1—C9—C10—C4	−1.07 (17)
C9—C1—C2—O2	177.39 (10)	C8—C9—C10—C4	178.91 (11)
C11—C1—C2—O2	−4.15 (17)	C1—C9—C10—C5	179.46 (11)
C9—C1—C2—C3	−2.75 (19)	C8—C9—C10—C5	−0.56 (18)
C11—C1—C2—C3	175.71 (11)	C2—C1—C11—O1	105.40 (14)
O2—C2—C3—C4	179.84 (12)	C9—C1—C11—O1	−76.15 (15)
C1—C2—C3—C4	0.0 (2)	C2—C1—C11—C12	−75.42 (14)
C2—C3—C4—C10	2.2 (2)	C9—C1—C11—C12	103.03 (13)
C10—C5—C6—C7	−1.5 (2)	O1—C11—C12—C13	165.98 (11)
C19—O3—C7—C8	−8.7 (2)	C1—C11—C12—C13	−13.19 (16)
C19—O3—C7—C6	171.37 (12)	O1—C11—C12—C17	−12.62 (17)
C5—C6—C7—O3	179.62 (12)	C1—C11—C12—C17	168.21 (10)
C5—C6—C7—C8	−0.3 (2)	C17—C12—C13—C14	1.17 (16)
O3—C7—C8—C9	−178.27 (12)	C11—C12—C13—C14	−177.45 (10)
C6—C7—C8—C9	1.65 (19)	C12—C13—C14—C15	−1.44 (17)
C2—C1—C9—C8	−176.75 (11)	C20—O4—C15—C14	4.39 (16)
C11—C1—C9—C8	4.82 (18)	C20—O4—C15—C16	−174.64 (10)
C2—C1—C9—C10	3.23 (17)	C13—C14—C15—O4	−178.69 (10)
C11—C1—C9—C10	−175.20 (11)	C13—C14—C15—C16	0.29 (17)
C7—C8—C9—C1	178.80 (11)	O4—C15—C16—C17	−179.82 (10)
C7—C8—C9—C10	−1.18 (18)	C14—C15—C16—C17	1.10 (17)
C3—C4—C10—C5	177.77 (13)	C15—C16—C17—C12	−1.38 (18)
C3—C4—C10—C9	−1.68 (19)	C13—C12—C17—C16	0.26 (17)
C6—C5—C10—C4	−177.53 (13)	C11—C12—C17—C16	178.92 (11)

### Hydrogen-bond geometry (Å, º)

**Table:**

*D*—H···*A*	*D*—H	H···*A*	*D*···*A*	*D*—H···*A*
C6—H6···O3^i^	0.95	2.51	3.4592 (16)	178

Symmetry code: (i) −*x*, −*y*, −*z*.
